# How Reusable Are Public Proteomics Datasets in Practice?

**DOI:** 10.1002/pmic.70175

**Published:** 2026-09-01

**Authors:** Giuseppe G. F. Leite, Victor Corasolla Carregari, Alexandre Keiji Tashima, Reinaldo Salomão

**Affiliations:** ^1^ Division of Infectious Diseases Department of Medicine Escola Paulista de Medicina Universidade Federal de Sao Paulo São Paulo Brazil; ^2^ Department of Laboratory and Hematological Sciences Unit of Chemistry Biochemistry and Molecular Biology “A. Gemelli” Hospital Foundation IRCCS Rome Italy; ^3^ Department of Biochemistry Escola Paulista de Medicina Universidade Federal de Sao Paulo São Paulo Brazil; ^4^ Laboratory of Mass Spectrometry Applied to Biomolecules Escola Paulista de Medicina Universidade Federal de Sao Paulo São Paulo Brazil

**Keywords:** FAIR proteomic data, metadata, processed quantitative outputs

## Abstract

Public proteomics repositories have expanded rapidly, but large‐scale reuse still depends on whether deposited studies are computationally reusable. We provide an evidence‐based snapshot of this gap by evaluating 500 non‐redundant human ProteomeXchange datasets released in 2025. Experimental design annotation was classified as metadata‐based when a deposited table (e.g., SDRF, spreadsheet, or text table) explicitly linked samples, files, channels, or quantitative columns to biological groups; sample‐based when groups could only be inferred from sample, column or channel names; and no information when reliable group assignment was not possible. Sample‐based datasets represented the largest category, comprising 195 deposits (39.0%), followed by datasets lacking reliable design information (170; 34.0%) and metadata‐based datasets (135; 27.0%). Among metadata‐based deposits, 81 (60.0%) included additional contextual covariates. Processed quantitative outputs were fully available in 245 datasets (49.0%), partially available in 168 (33.6%), and absent in 87 (17.4%). Combining processed quantitative outputs with available biological design information, 189 datasets (37.8%) supported direct matrix‐level reuse. Under a stricter scenario requiring metadata‐based design, contextual covariates and available processed quantitative outputs, only 64 datasets (12.8%) fulfilled all criteria. These findings suggest that scalable reuse requires more consistent machine‐actionable sample‐to‐file mapping, interpretable design annotation, processing information and processed outputs, while preserving raw data for harmonized reanalysis.

## 1 Introduction

Proteomics has expanded rapidly over the past decade, becoming a central pillar of biomedical research and generating an unprecedented volume of publicly available data. Between 2020 and 2025, we identified 40,826 datasets that are publicly available through the ProteomeXchange consortium [1]. These datasets originate from multiple repositories, including PeptideAtlas and PASSEL [2], Panorama Public [3], MassIVE [4], PRIDE database [5], iProX [6], and jPOST [7].

In parallel, the community has developed standards aimed at improving transparency, reproducibility, and data reuse in proteomics. A major advance in this direction is the introduction of the MAGE‐TAB‐Proteomics format [8], an extension of the original MAGE‐TAB framework developed for transcriptomics [9], designed to support structured metadata annotation of proteomics datasets. MAGE‐TAB‐Proteomics consists of two core components: (i) the Investigation Description Format (IDF), which captures study‐level descriptors, and (ii) SDRF‐Proteomics, a tab‐delimited format that explicitly maps biological samples to mass‐spectrometry data files and encodes experimental factors [10].

Prior to the development of MAGE‐TAB‐Proteomics, complementary efforts such as MassIVE.quant were established to support the sharing of quantitative proteomics data, metadata, and reanalyses [11]. In parallel, jPOST implemented unified reanalysis workflows, which revealed the need for substantial improvements in the collection, completeness, and structuring of experimental metadata [12]. Together, these efforts indicate that expectations for structured metadata and reusable outputs in proteomics are well defined at the community level.

Despite this, barriers to reproducibility and data reuse remain widespread. Inconsistent adoption and partial implementation of existing standards continue to limit the practical reusability of public proteomics datasets. In many cases, essential information is omitted or encoded in forms that do not support scalable reanalysis, effectively shifting the burden of interpretation and reconstruction to downstream users.

Rather than proposing new standards, we take a diagnostic stance and ask a simpler question: how well are existing expectations translated into routine practice, as experienced by a secondary user who has access only to repository‐deposited materials?

## 2 What Does “REUSABLE” Means in Practice?

In practice, proteomics reuse depends first on whether the experimental design can be interpreted without guesswork. Users need to understand how samples, files, channels, and biological groups relate to one another before either raw‐data reanalysis or matrix‐level analysis can be performed reliably.

Additional contextual information, such as batch, sex, age, disease severity, or technical variables, can further improve reuse by enabling adjusted analyses and assessment of confounding. Raw data remain essential for standardized reanalysis, especially when users need to harmonize databases, search parameters, quantification algorithms, or normalization strategies across studies. However, processed quantitative outputs simplify reuse by allowing direct downstream analysis in environments such as R or Python, provided that the experimental design is interpretable.

## 3 An Evidence‐Based Snapshot of 2025 Deposits

To ground this Viewpoint in empirical evidence, we queried the ProteomeXchange portal on December 9, 2025 for *Homo sapiens* datasets released in 2025, excluding entries flagged as “publication pending” or “no publication,” as these deposits often have incomplete public releases at the time of submission. From the resulting list (sorted by publication date), we screened datasets from newest to oldest and continued until we obtained a non‐redundant working set of 500 deposits. Redundancy was handled during selection: near‐duplicate deposits that were clearly linked to the same underlying study were removed and additional older entries were screened to maintain a final sample size of 500. The resulting set spanned announcements from December 9 back to August 29, 2025. Each dataset was evaluated using only repository‐deposited files and descriptions, without consulting associated manuscripts, supplementary PDFs, or external websites — reflecting realistic reuse conditions. Dataset screening was performed manually. One reviewer classified all datasets and subsequently re‐evaluated the complete dataset in a second screening pass conducted at a different time before finalizing the classifications. To assess classification consistency, a second reviewer independently evaluated a random subset of datasets. Discrepancies were resolved through discussion until consensus was reached.

All accessions and classification outcomes are provided in Supporting Information 1 to facilitate independent verification and alternative reanalysis of our screening decisions. We evaluated three indicators directly relevant to data reuse: experimental design clarity, availability of additional contextual information, and processed data availability. The screening and classification procedure is summarized as a decision workflow in Figure 1.

**FIGURE 1:**
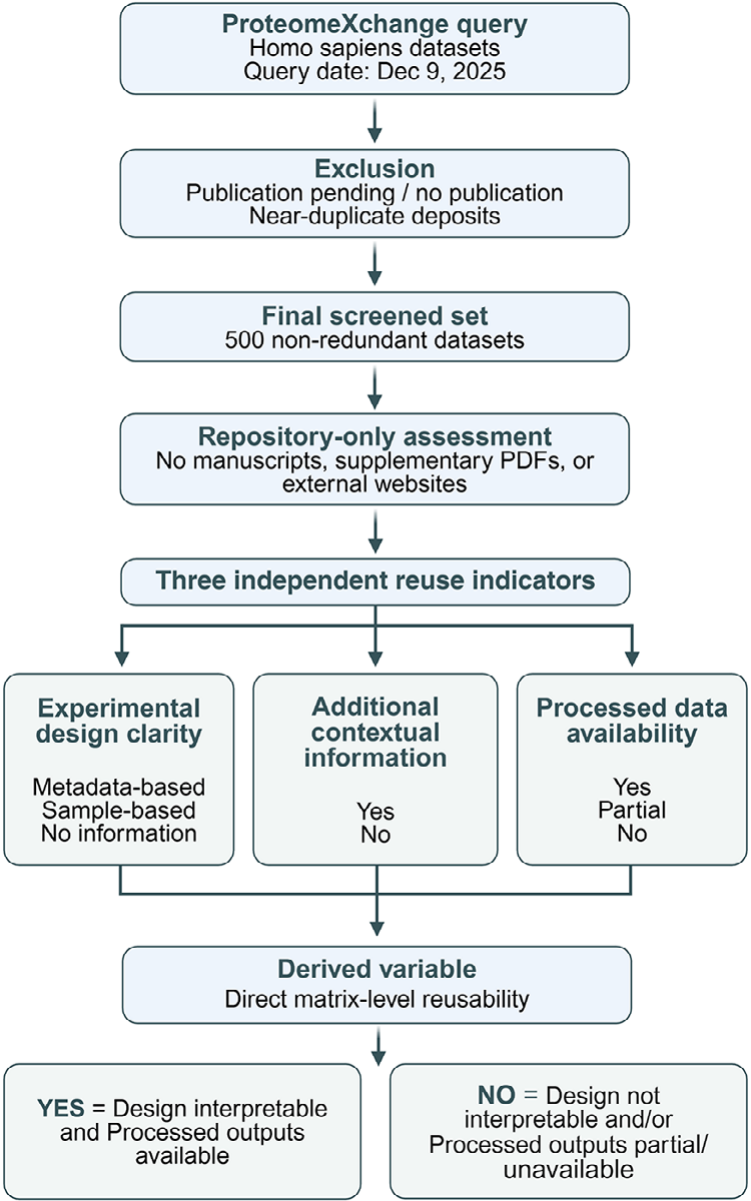
Workflow for screening and assessment of ProteomeXchange datasets. The diagram summarizes the repository‐only screening strategy, exclusion criteria, reuse indicators, and derivation of direct matrix‐level reusability. Created in BioRender. Salomao, R. (2026) https://BioRender.com/khikkfv.

With respect to experimental design clarity, datasets were classified as “metadata‐based” when a deposited metadata file (e.g., SDRF, spreadsheet, or text table) explicitly linked biological samples to raw data files and enabled unambiguous reconstruction of biological group structure (e.g., case–control status, treatment arms, genotype, or time point). For multiplexed or column‐based quantitative outputs, this criterion also required that channels, labels, files, or quantitative columns could be linked to the corresponding biological samples or experimental groups. Datasets were classified as “sample‐based” when group structure could be inferred only from sample names, file names, or quantitative column names in the absence of a deposited mapping table. Datasets were classified as “no information” when neither deposited metadata nor interpretable naming conventions supported reliable group assignment.

For datasets classified as “metadata‐based,” we further recorded whether additional contextual covariates were provided (e.g., sex, age, disease severity, batch effects, or technical variables).

Independently of experimental design clarity, we assessed processed data availability. Datasets were classified as “yes” when they contained processed quantitative outputs in a tabular format, such as protein‐, peptide‐, or feature‐level quantitative matrices generated by commonly used software including MaxQuant, DIA‐NN, Spectronaut, or Proteome Discoverer. Datasets were classified as “partial” when processed outputs were present but were incomplete, constrained by proprietary formats or software, not directly usable as a conventional quantitative matrix, or insufficiently documented to determine whether they represented final quantitative outputs. Datasets were classified as “no” when deposits contained only raw or intermediate files, such as RAW, mzML, MGF, search‐engine outputs, or identification‐only files, and lacked processed quantitative outputs.

To summarize whether deposited datasets could be directly reused in general‐purpose computational environments, we derived a binary variable termed “direct matrix‐level reusability.” Datasets were classified as “yes” when they had interpretable experimental design, either metadata‐based or sample‐based, and available processed quantitative outputs. Datasets were classified as “no” when processed data were unavailable or only partially available, or when the biological group structure could not be reliably reconstructed.

## 4 Data Completeness Remains Uneven Across Public Proteomics Deposits

Across the 500 evaluated datasets, sample‐based experimental design annotation was the most frequent category (195; 39%), followed by datasets with no reliable experimental design information (170; 34%). Only 135 datasets (27%) provided metadata‐based experimental design annotations (Figure 2A). Among metadata‐based datasets, 60% (*n* = 81) included additional contextual covariates, including sex, age, disease severity, batch structure, or technical variables (Supporting Information 1). By contrast, sample‐based and no‐information datasets typically lacked contextual detail beyond that inferred from sample, file, column, or channel naming conventions.

**FIGURE 2:**
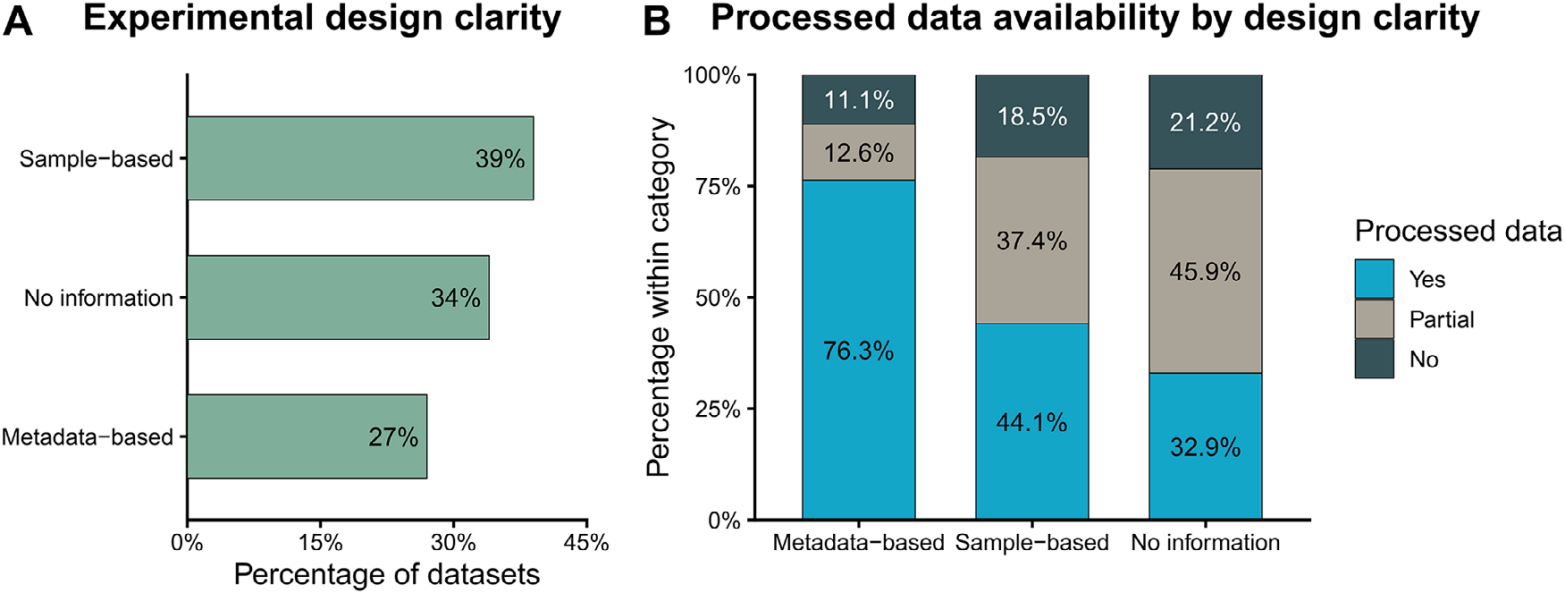
Repository‐level classification of 500 ProteomeXchange datasets.(A) Experimental design clarity was classified as metadata‐based when deposited metadata enabled reconstruction of biological group structure, sample‐based when group structure could be inferred from sample, file, column, or channel naming, and no information when reliable group assignment was not possible. Counts were: metadata‐based, *n* = 135 (27.0%); sample‐based, *n* = 195 (39.0%); no information, *n* = 170 (34.0%). (B) Processed data availability was assessed independently within each experimental design category and classified as yes, partial, or no according to the availability of processed quantitative outputs in tabular format.

Processed quantitative outputs were available in 245 of 500 datasets (49.0%), partially available in 168 datasets (33.6%), and absent in 87 datasets (17.4%). Availability differed substantially by experimental design category (Figure 2B). Processed data were most frequent among metadata‐based deposits (76.3%) and decreased in sample‐based deposits (44.1%) and deposits with no design information (32.9%). The opposite pattern was observed for partial or absent processed data, which increased as design annotation became less informative.

We therefore distinguished processed data availability from direct matrix‐level reusability. Processed quantitative outputs alone were not sufficient for direct reuse unless the biological design could also be reconstructed. Using this derived criterion, 189 of 500 datasets (37.8%) were directly reusable at the matrix level. Under the most stringent reuse scenario, requiring metadata‐based design annotation, additional contextual information, and available processed quantitative outputs, only 64 datasets (12.8%) fulfilled all criteria.

## 5 From Public Availability to Computable Reuse

Our 2025 snapshot indicates that proteomics data sharing is improving, but that public availability still does not necessarily translate into computable reuse. In FAIR (Findable, Accessible, Interoperable, Reusable) terms, the limitation is not only whether files are accessible, but whether deposited data are sufficiently findable, interoperable and reusable by both humans and machines [13, 14]. This distinction is critical in proteomics, where reuse depends on explicit relationships among biological samples, raw files, multiplexed channels, fractions, quantitative columns, experimental groups, and relevant covariates [8, 10].

The 27% of deposits classified here as metadata‐based should be viewed as an encouraging signal. Previous PRIDE‐focused work reported that only 1.6% of eligible datasets contained submitter‐supplied SDRF annotation, increasing to 3.9% when post hoc community annotation was included [15]. Although our estimate is not directly comparable, because we also counted deposited spreadsheets or text tables that enabled reconstruction of the experimental design, it suggests a positive shift toward more interpretable submissions. Nevertheless, even under this broader definition, metadata‐based annotation remained a minority of deposits. Thus, current practice appears to be improving, but structured metadata have not yet become routine.

The main barrier identified in our assessment was not simply missing metadata, but missing computable metadata. Information inferred from filenames, free‐text repository descriptions, column labels, or scattered supplementary files may be understandable to a human reader, but it cannot be reliably parsed by downstream workflows. This is especially problematic for multiplexed and fractionated experiments, where sample‐to‐file relationships are not necessarily one‐to‐one [16]. As a result, users may need to manually reconstruct group labels, sample‐to‐file mappings or TMT channel assignments, introducing ambiguity and limiting reproducible reanalysis.

These findings suggest that the field does not lack standards; rather, their implementation remains uneven. SDRF‐Proteomics, ProteomeXchange/PSI standards and MAGE‐TAB‐based sample metadata models already provide mechanisms to encode experimental design and sample‐to‐data relationships in structured formats [8, 10, 17]. However, standards alone are insufficient if metadata creation remains a retrospective task performed only at submission. Metadata should be generated, validated, and propagated during analysis. Recent SDRF support in tools such as MaxQuant illustrates how analysis software can reduce the burden on users by exporting structured metadata and annotating output tables directly [16].

A practical implication is that reusable proteomics deposits should include, at minimum, raw files, processed quantitative outputs, and a structured sample‐to‐data map linking samples, files, channels, fractions, groups, and core covariates. Achieving this will require coordinated action from repositories, software developers, data producers, journals, and reviewers [18, 19, 20]. Public proteomics has largely succeeded in making data available; the next challenge is to make those data routinely computable.

## 6 Processed Data Lower the Barrier to Reuse, but do not Define Reusability Alone

Our data show that processed quantitative outputs were available in nearly half of the deposits, but were unevenly distributed across design categories. Their availability increased with the quality of experimental design annotation: deposits with metadata‐based design information were more likely to include complete processed data, whereas deposits with less informative design annotation more often provided only partial outputs or raw data. This pattern indicates that processed data availability and interpretable design often co‐occur, but are not equivalent. Several deposits provided quantitative outputs without sufficient design information to support direct matrix‐level reuse.

Processed outputs lower the barrier to secondary analysis, from targeted inspection of proteins of interest to broader cross‐dataset reanalysis. When quantitative matrices are linked to sample annotations and group information, they can be analyzed directly; without these links, their biological value is limited because groups and contrasts cannot be defined [16, 19, 21]. However, this form of reuse still depends on processing context, including database or library choice, normalization, imputation, and workflow settings, particularly in meta‐analyses [17, 21, 22].

Raw files support a complementary reuse scenario. Whereas processed outputs enable direct analysis of deposited quantitative results, raw files allow expert users to reprocess datasets under harmonized conditions. This is important for cross‐study integration, benchmarking, and large‐scale reanalysis, where datasets may need to be searched and quantified using consistent FASTA databases, spectral libraries, search parameters, quantification algorithms, and statistical workflows. Resources such as MassIVE.quant and quantms illustrate how standardized reprocessing can improve consistency and comparability across public datasets [17, 21]. This strategy is essential for expert reanalysis, but requires computational infrastructure, domain expertise and processing time [23].

Taken together, processed and raw data serve different reuse needs. Processed outputs increase accessibility for downstream biological analysis, whereas raw data enable harmonized reprocessing and methodological standardization. Robust reuse is best supported when deposits include accessible processed outputs together with sufficient contextual and processing information to understand or reproduce how those outputs were generated.

## 7 Why Did Transcriptomics Scaled Reuse Faster—And Why Does Proteomics Differs?

Transcriptomics is a useful comparator because its public repositories made deposited data relatively easy to convert into structured inputs for downstream analysis. In Gene Expression Omnibus (GEO), expression measurements are typically distributed together with sample‐level descriptions, and GEOquery made these records directly accessible from the Bioconductor environment [24, 25]. This created a low‐friction route from data discovery to statistical analysis: users could retrieve expression values, align them with sample annotations and begin downstream modeling with limited manual restructuring.

The comparison is informative, but not symmetrical. Transcriptomic data can often be represented as a sample‐by‐gene table with relatively stable feature definitions. Mass‐spectrometry proteomics is more heterogeneous: datasets differ in acquisition mode, labeling strategy, fractionation, enrichment protocol, protein post‐translational modifications, instrument platform, search engine, protein inference, and quantification approach [8, 22]. In multiplexed or fractionated experiments, one biological sample may map to several files, and one file may contain several samples. These features make proteomics reuse more dependent on methodological reconstruction than transcriptomics, even when quantitative outputs are available [8, 21, 22].

This technical asymmetry helps explain why transcriptomics datasets have historically been reused more often than proteomics datasets [21]. The lesson from transcriptomics is therefore not that proteomics should adopt an identical model, but that reuse scales when repository outputs match the analytical structure of the field. In proteomics, scalable reuse will likely depend on linking interpretable sample and design annotation to infrastructure that can accommodate heterogeneous acquisition strategies, specialized workflows and standardized reprocessing where needed. Such an approach would allow public proteomics data to move beyond archival availability toward more cumulative reuse.

## 8 Conclusions

Public proteomics data deposition is growing rapidly, but reuse at scale remains limited by incomplete machine‐actionable information and workflow heterogeneity. In our 2025 snapshot, many deposits lacked either interpretable experimental design information or processed quantitative outputs sufficient to support direct matrix‐level reuse, while broader reanalysis also depends on access to raw data and processing information. This gap creates practical selection effects, where computational feasibility—rather than biological relevance alone—can influence which datasets are reused. The main need is not necessarily new standards, but more consistent adoption and enforcement of existing ones, together with repository and software infrastructure that preserves raw data, sample‐to‐file mapping, essential covariates, processing information, and processed quantitative outputs at submission.

## 9 Limitations

Our analysis is based on a non‐exhaustive snapshot of 500 recent ProteomeXchange deposits rather than the full public record. While this sample was selected to reflect realistic reuse conditions, it may not capture all patterns present across the complete dataset universe. In addition, our framework focuses on direct matrix‐level reusability, which is most applicable to quantitative proteomics studies designed for comparative downstream analysis. It may therefore underestimate the reusability of non‐matrix‐centric datasets, such as interactomics, immunopeptidomics, cross‐linking MS, de novo sequencing or selected post‐translational modification workflows, where reuse depends on specialized outputs rather than conventional quantitative matrices. Although all classifications were re‐evaluated in a second screening pass by the same reviewer, the complete dataset was not independently assessed by a second reviewer. An independent review of a random subset of 122 datasets showed 98.4% agreement, supporting the consistency of the classification framework.

## Author Contributions

G.G.F.L. and R.S. conceptualized and planned the viewpoint. G.G.F.L. collected the data, performed the primary dataset classification, and performed the data visualization. V.C.C. performed the independent secondary dataset screening. G.G.F.L. wrote the original draft of the manuscript. V.C.C. contributed to writing and reviewing the manuscript. R.S. and A.K.T. critically reviewed and edited the manuscript. All authors approved the final version.

## Funding

This work was supported by the São Paulo Research Foundation (FAPESP), Grant Number. 24/01786‐2, awarded to R.S. G.G.F.L. currently receives a scholarship from FAPESP (Grant Number. 2025/20381‐6).

The Article Processing Charge for the publication of this research was funded by the Coordenação de Aperfeiçoamento de Pessoal de Nível Superior ‐ Brasil (CAPES) (ROR identifier: 00x0ma614).

## Conflicts of Interest

The authors declare no conflicts of interest.

## Supporting information




**Supporting File**: pmic70175‐sup‐0001‐SuppMat.zip.

## Data Availability

The accession list of the 500 screened datasets and all classification outcomes are provided in Supporting Information Table 1.
